# Apocynin prevents cigarette smoking‐induced loss of skeletal muscle mass and function in mice by preserving proteostatic signalling

**DOI:** 10.1111/bph.15482

**Published:** 2021-06-08

**Authors:** Stanley M. H. Chan, Ivan Bernardo, Chanelle Mastronardo, Kevin Mou, Simone N. De Luca, Huei Jiunn Seow, Aleksandar Dobric, Kurt Brassington, Stavros Selemidis, Steven Bozinovski, Ross Vlahos

**Affiliations:** ^1^ School of Health and Biomedical Sciences RMIT University Melbourne Victoria Australia

**Keywords:** antioxidants, chronic obstructive pulmonary disease, IGF‐1, NADPH oxidase, protein carbonylation

## Abstract

**Background and Purpose:**

Skeletal muscle dysfunction is a major comorbidity of chronic obstructive pulmonary disease (COPD). This type of muscle dysfunction may be a direct consequence of oxidative insults evoked by cigarette smoke (CS) exposure. The present study examined the effects of a potent Nox inhibitor and reactive oxygen species (ROS) scavenger, apocynin, on CS‐induced muscle dysfunction.

**Experimental Approach:**

Male BALB/c mice were exposed to either room air (sham) or CS generated from nine cigarettes per day, 5 days a week for 8 weeks, with or without the coadministration of apocynin (5 mg·kg^−1^, i.p.). C2C12 myotubes exposed to either hydrogen peroxide (H_2_O_2_) or water‐soluble cigarette smoke extract (CSE) with or without apocynin (500 nM) were used as an experimental model in vitro.

**Key Results:**

Eight weeks of CS exposure caused muscle dysfunction in mice, reflected by 10% loss of muscle mass and 54% loss of strength of tibialis anterior which were prevented by apocynin administration. In C2C12 myotubes, direct exposure to H_2_O_2_ or CSE caused myofibre wasting, accompanied by ~50% loss of muscle‐derived insulin‐like growth factor (IGF)‐1 and two‐fold induction of *Cybb*, independent of cellular inflammation. Expression of myostatin and MAFbx, negative regulators of muscle mass, were up‐regulated under H_2_O_2_ but not CSE conditions. Apocynin treatment abolished CSE‐induced *Cybb* expression, preserving muscle‐derived IGF‐1 expression and signalling pathway downstream of mammalian target of rapamycin (mTOR), thereby preventing myofibre wasting.

**Conclusion and Implications:**

Targeted pharmacological inhibition of Nox‐derived ROS may alleviate the lung and systemic manifestations in smokers with COPD.

## Graphical abstract

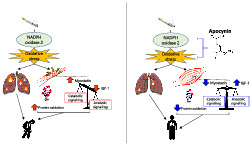

**Abbreviations**
4E‐BP1: eukaryotic translation initiation factor 4E‐binding protein 1
BALF: bronchoalveolar lavage fluid
COPD: chronic obstructive pulmonary disease
CS: cigarette smoking
CSE: cigarette smoke extract
DM: differentiation medium
eIF2α: eukaryotic translation initiation factor 2A
FoxO: forkhead box class O
GMCSF: granulocyte–macrophage colony‐stimulating factor
Gpx: glutathione peroxidase
IGF: insulin‐like growth factor
LC3: autophagy marker Light Chain 3
Lo: optimal muscle length
MAFbx: muscle atrophy F‐box
Mstn: myostatin
MuRF: muscle RING finger 1
Nox: nicotinamide adenine dinucleotide phosphate oxidase
O_2_^‐^
: superoxide anion
PBST: phosphate‐buffered saline with Tween 20
Pt: peak twitch force
ROS: reactive oxygen species
TA: tibialis anterior muscle
UPS: ubiquitin‐proteasome system

**What is already known**

- Skeletal muscle dysfunction is a major COPD comorbidity that predicts disease morbidity and mortality.
- Skeletal muscle dysfunction may be evoked by oxidative stress from cigarette smoke (CS) exposure.

**What this study adds**

- By disrupting myogenic homeostasis, CS‐induced oxidative stress causes skeletal muscle dysfunction in mice.
- Apocynin preserved muscle mass and function against the detrimental oxidative effects of CS exposure

**Clinical significance**

- Targeting oxidative stress may improve pulmonary and systemic outcomes associated with COPD.

## 1 INTRODUCTION

Chronic obstructive pulmonary disease (COPD) is characterised by progressive airflow limitation that is not fully reversible (Vogelmeier et al., 2017). Cigarette smoking (CS) is the major cause of COPD accounting for 80–90% of cases in industrialised countries (Vogelmeier et al., 2017). In addition to the pulmonary pathologies, COPD may also give rise to debilitating conditions (i.e., comorbidities) in extra‐pulmonary tissues which may lead to a deterioration of function, quality of life and mortality (Fabbri & Rabe, 2007). Skeletal muscle dysfunction is one of the most common comorbidities that affects up to 40% of COPD patients (Passey et al., 2016). Skeletal muscle dysfunction limits exercise performance and capacity, thereby is detrimental to the overall health of those suffering from COPD, irrespective of decreased lung function (Swallow et al., 2007). In addition, muscle dysfunction may be a predictor of future acute exacerbations and hospital readmission (Vilaro et al., 2010), suggesting skeletal muscle function may be a determinant of health outcomes in COPD patients.

Muscle dysfunction marks the inability of a muscle to perform its task, leading to the manifestation of muscle weakness and fatigue (Yamano et al., 2010). By definition, muscle weakness (i.e., loss of strength) and fatigue (i.e., loss of endurance) are distinct conditions but the observation that a weak muscle becomes more easily fatigued has highlighted the inseparable nature of the two defects (Yamano et al., 2010). Indeed, both reduced force‐generating capacity and fatigue resistance have been observed in limb muscles of COPD patients, leading to exercise intolerance (Vogelmeier et al., 2017).

The observations that even a single bout of smoking was sufficient to decrease exercise capacity (Hirsch et al., 1985), and that muscle dysfunction may be found in non‐symptomatic smokers (Corwin et al., 2002), have led to the concept that CS may directly affect muscle function. In non‐symptomatic smokers and patients with COPD, Barreiro et al. (Barreiro et al., 2010) demonstrated that CS exposure directly elicits oxidative stress in the vastus lateralis muscle which may contribute to atrophy and dysfunction. Importantly, the same study also reported no significant rise in muscle inflammation amongst smokers and COPD patients, thus confirming the direct effects of CS exposure on muscle dysfunction which may be exerted through an oxidative stress‐driven mechanism, that is independent of inflammation.

Nicotinamide adenine dinucleotide phosphate (NADPH) oxidase (Nox) is a multimeric enzyme that catalyses the formation of reactive oxygen species (ROS) and the superoxide anion (O_2_^−^), which is the parent species that ultimately contributes to oxidative stress (Griffith et al., 2009). Not only is O_2_^−^, in itself, a potent oxidant, but it can be converted into hydrogen peroxide (H_2_O_2_ ), which is a more influential form of ROS in terms of redox signalling with a longer half‐life (Griffith et al., 2009). The role of Nox‐derived ROS has long been recognised in the pathogenesis of COPD. However, deletion of Cybb (the gene encoding Nox2) or its catalytic subunit, p47^phox^, was found to incur higher levels of lung inflammation and alveolar destruction in mice exposed to CS, despite decreasing ROS production (Yao et al., 2008). This suggests normal expression of Nox2 is essential for maintaining redox and immune homeostasis.

In skeletal muscle, functional Nox enzyme complexes have been detected at the plasma membrane during muscular contraction, suggesting its active involvement in muscle function (Sakellariou et al., 2013). However, the exact role of Nox‐derived ROS in CS‐induced muscle dysfunction remains unclear. Given the detrimental effects of genetic disruption of Nox, the present study adopted a pharmacological approach, using apocynin. Apocynin inhibits Nox activation by blocking the cytosolic to membrane translocation of p47^phox^ and p67^phox^, thereby disrupting the assembly of the active enzyme complex (Johnson et al., 2002). Furthermore, apocynin has also been shown to act as a scavenger for O_2_^−^ and other ROS (Heumuller et al., 2008). The present study examined whether inhibition of Nox‐derived ROS would attenuate lung inflammation and muscle dysfunction induced by CS exposure.

## 2 METHODS

### 2.1 Mice

All animal care and experimental procedures were conducted in accordance with the Australian Code of Practice for the Care of Experimental Animals and the ARRIVE Guidelines, and were apporived by the RMIT University Animal Ethics Committee (Animal Ethics Application Number 1521). Male BALB/c mice (RRID:IMSR_ORNL:BALB/cRl, 7 weeks of age) were obtained from the Animal Resource Centre (Perth, Australia). Mice were housed in micro‐isolator cages at 21°C on a 12‐h day/night cycle with free access to food and water.

After 4 days of acclimatisation, mice were randomly assigned to room air (sham) or cigarette smoke (smoke) exposure groups, with or without daily supplementation of apocynin (5 mg·kg^−1^), given i.p.. The vehicle groups were injected with saline (solvent of apocynin). Mice were weighed three times a week with daily monitoring. The animal experiments were independently performed twice with all four experimental groups (i.e., Sham vehicle, Sham apocynin, Smoke vehicle & Smoke apocynin) with n = 8–10 mice per group.

### 2.2 CS exposure and muscle function analysis

Mice were placed in 18 L perspex chambers and exposed to CS from three cigarettes (Winfield Red, 16 mg or less of tar, 15 mg or less of carbon monoxide, 1.2 mg or less of nicotine) spaced evenly over 1 h and carried out three times per day (09:00, 12:00, and 15:00 h), 5 days a week (Monday to Friday) for 8 weeks. The sham mice were handled identically and exposed to room air. We have previously shown that this CS exposure protocol in male Balb/C mice replicates key clinical traits of human COPD, including lung inflammation and pathology (emphysema, mucous hypersecretion, impaired lung function), increased lung and systemic oxidative stress and comorbidities including skeletal muscle dysfunction (Austin et al., 2016; Chan et al., 2020; Vlahos & Bozinovski, 2014).

At the end of the exposure protocol, in situ muscle function analysis was performed as previously described (Chan et al., 2020). In brief, mice were anaesthetised with ketamine (80 mg kg^‐1^) / xylazine (16 mg kg^‐1^) and small incisions were then made on the skin to expose the tibialis anterior (TA) muscle taking care not to damage the fascia. The mouse was secured on the heated platform (37°C) of an in situ contractile apparatus (809B in situ Mouse Apparatus, Aurora Scientific, Canada) with a pin behind the patellar tendon and a foot clamp. The distal end of the TA was tied firmly to a lever arm attached to an isometric force transducer. Two fine electrodes (3–5 mm apart) were inserted into the belly of the TA muscle. The muscle was stimulated by two field stimulating platinum electrodes coupled to an amplifier. The TA muscle was contracted via square wave (0.2 ms) pulses at 10 V from the stimulator (701C stimulator, Aurora Scientific, Canada). Forces were converted to a digital signal and recorded by DYNAMIC MUSCLE ANALYSIS 611A™ (Aurora Scientific, Canada). Optimum muscle length (Lo) was first determined by eliciting twitch contractions by incrementally adjusting muscle length with a micromanipulator until a repeatable maximum peak twitch force was obtained. Optimal muscle length (L_o_) was measured with precision digital callipers from the beginning of the distal tendon to the insertion of the TA at the base of the knee. Subsequently, the TA was stimulated at 100 Hz tetanic contraction, followed by a 2 min rest interval, and then twitch contraction. Comparable twitch forces pre and post 100 Hz stimulation indicated that the knots were both secure and unlikely to slip during the remaining protocol. If a decrease in twitch force was observed, the muscle was incrementally tensioned and stimulated between 2 min rest intervals until peak twitch force (Pt) was re‐established. To establish the force frequency relationship, the TA was stimulated supramaximally (10 V) for 500 ms at 10, 20, 30, 40, 50, 80, 100, 150, 200, 250 and 300 Hz, with a 2 min rest interval in between.

To delineate whether the observed muscle weakness induced by CS was attributable to reduced muscle mass and/or an impaired excitation–contraction coupling, the maximal contractile force at 120 Hz is normalised to the whole‐muscle cross sectional area to produce the specific muscle force. The cross sectional area can be approximated from the gross mass and L_0_ of the muscle, together with the muscle density (~1.06 g·cm^−3^) (Close, 1972). Hence, the following equation is used:

$$\text{Specific}\;\;\;\text{force}=\frac{\text{Maximal}\;\;\text{contractile}\;\;\;\text{force}\;\;\;\mathrm{at}\;120\;\mathrm{Hz}\;\left(\mathrm{mN}\right)}{\text{Muscle}\;\;\;\text{mass}\;\left(g\right)/1.06\;\left(g\cdot cm^{-3}\right)\times L_{o}\;\left(\mathrm{cm}\right)\;}$$

Immediately following the contraction protocol, the mouse was removed from the apparatus and killed with an overdose of anaesthetic (sodium pentobarbitone; 240 mg kg^‐1^, i.p.) to continue with tissue collection and the procedures described below. Muscle mass was measured using an analytical balance after the tendons and other non‐muscle tissues were removed and after brief contact with absorbent material such as filter paper to remove excess solution.

### 2.3 Tissue collection

The lungs were lavaged in situ using 0.4 ml of ice‐cold phosphate‐buffered saline (PBS) and three subsequent repeats of 0.3 ml PBS, with a return of approximately 1 ml of bronchoalveolar lavage fluid (BALF) per mouse, as previously published (Chen et al., 2006; Vlahos et al., 2006). Twenty microlitres of BALF was diluted 1:1 with Acridine Orange and the total number of viable cells counted on a standard Neubauer haemocytometer under fluorescent light on an Olympus BX53 microscope (Olympus, Japan). To differentiate cell populations in BALF, cytocentrifuge preparations (Shandon Cytospin 3, 18 x *g*, 10 min) were performed using approximately 5 × 10^4^ cells from BALF. Once dried, cells were fixed with Shandon™ Kwik‐Diff™ fixative (Thermo Fischer Scientific, USA) and subsequently stained with Hemacolor® Rapid Red and Blue dye (Merck, Germany) according to the manufacturers' instructions, mounted with Enetellan® new (Merck, Australia). Cell types (macrophages, lymphocytes and neutrophils) were identified according to standard morphological criteria. At least 500 cells per slide were counted. After the lavage procedure, 10 ml of PBS was used to clear the lungs of blood via a right ventricular perfusion of the heart. Lungs were then weighed, snap frozen in liquid nitrogen and stored at −80°C until required. Lower limb muscles were removed tendon to tendon from each mouse. The muscles were weighed, snap frozen in liquid nitrogen and stored at −80°C until required.

### 2.4 Quantitative real‐time polymerase chain reaction

Total RNA was extracted from tissues and cultured myotubes using RNeasy kits (Qiagen, USA), reverse transcribed using High‐Capacity RNA‐to‐cDNA™ Kit (Thermo Fisher Scientific, USA) before real‐time polymerase chain reaction (PCR) analysis using QuantStudio 7™ (Thermo Fisher Scientific, USA). All reactions were performed in triplicate using Taqman Fast Advanced Master Mix and pre‐developed gene expression assays (Table 1) except for *Igf1‐eb* (Thermo Fisher Scientific) and data obtained were normalised against GAPDH as reference gene prior to analysis using the ΔΔCT method, as previously described (Vlahos et al., 2011).

**TABLE 1:** List of gene expression assays

Gene name	Abbreviation	Taqman assay ID
Granulocyte–macrophage colony‐stimulating factor 2	*Gmcsf*	Mm01290062_m1
Chemokine CCL2	*Ccl2*	Mm00441242_m1
Chemokine CXCL2	*Cxcl2*	Mm00436450_m1
Tumour necrosis factor	*Tnfα*	Mm00443258_m1
Insulin‐like growth factor Ea	*Igf‐ea*	Mm00710307_m1
Insulin‐like growth factor Eb	*Igf‐eb*	AIKALFT
Myostatin	*Mstn*	Mm01254559_m1
Cytochrome b‐245, beta polypeptide (NADPH oxidase 2)	*Cybb*	Mm01287743_m1
Glutathione peroxidase 1	*Gpx1*	Mm00656767_g1
Interleukin‐6	*Il‐6*	Mm00446190_m1
F‐box protein 32 (MAFbx)	*Fbxo32*	Mm00499523_m1

### 2.5 Cell culture and intervention protocols

C2C12 murine myoblasts (American Type Culture Collection, CRL‐1772, RRID:CVCL_0188) were cultured in growth medium consisting of Dulbecco's modified Eagle's medium (DMEM; Thermo Fisher Scientific, USA) supplemented with 1% penicillin/streptomycin (100 units ml^‐1^ penicillin and 100 μg ml^‐1^ streptomycin; Thermo Fisher Scientific, USA) and 10% foetal bovine serum (FBS; Thermo Fisher Scientific, USA). Cells were cultured in a T‐75 culture flask at a density of 5 × 10^3^ viable cells cm^‐2^ and were passaged at 70–80% confluence. Flasks were kept in a humidified incubator at 37°C with the supplementation of 5% CO_2_. To induce differentiation, confluent monolayers of C2C12 myoblasts were cultured in differentiation medium (DM) consist of DMEM supplemented with 1% penicillin/streptomycin and 2% horse serum (Thermo Fisher Scientific, USA) and the DM was changed daily. All experiments were performed on Day 6 when most myoblasts have fused to form mature myotubes.

The gas phase of CS, otherwise known as cigarette smoke extract (CSE), was prepared by bubbling the smoke from one cigarette (Winfield Red, Phillip Morris International) through 25 ml of pre‐warmed DM at a rate of 5 ml·s^−1^ to produce 100% CSE stock solution. The stock solution was sterile filtered and serially diluted with pre‐warmed DM to obtain concentrations required for experimentation. Hydrogen peroxide (H_2_O_2_) was prepared in sterile water resulting in a 3000 μM stock solution. The stock solution was serially diluted with pre‐warmed DM to obtain the required concentrations for experimentation. Apocynin was dissolved in DMSO to give a solution of 500 μM (stock solution). The stock solution was diluted with pre‐warmed DM to give a final concentration of 500 nM. To ensure bioavailability, apocynin was pre‐incubated for 30 min prior to administration of the respective oxidative insults (H_2_O_2_ or CSE), replicating those in our animal model.

### 2.6 Oxyblots and western blots

Approximately 20 mg of muscle tissue was homogenised in 500 μl of RIPA lysis buffer containing 1% protease inhibitors cocktail. For cell experiments, C2C12 myotubes in six‐well plates were collected and homogenised in 100 μl of RIPA lysis buffer. The samples were then centrifuged for 10 min at 14,000 × *g*, 4°C. The supernatant was collected for immediate use or stored at −80°C. Protein concentrations were determined with a commercially available colorimetric bicinchonnic acid (BCA) protein kit, to standardise the loading amount for the SDS‐PAGE. SDS‐PAGE was conducted as previously described (Chan et al., 2013) with specific antibodies against phospho‐eIF2α (Ser51, Cell Signaling Technology, USA #3398, RRID:AB_2096481), phospho‐S6 Ribosomal Protein (Ser235/236, Cell Signaling Technology, USA #4858, RRID:AB_331682), phospho 4E‐BP1 (Thr37/46, Cell Signaling Technology, USA #2855, RRID:AB_560835), Fbx32/MAFbx (Abcam, USA ab 10859967, RRID:AB_10859967), 19S proteasome (Abcam, USA ab2857944, RRID:AB_2857944), LC3A/B (Cell Signaling Technology, USA #12741, RRID:AB_2617131), p62 (Cell Signaling Technology, USA #23214, RRID:AB_2799160), actin (Cell Signaling Technology, USA #4968, RRID:AB_2313904).

For oxyblots, the extracted protein samples were derivatised and stabilised using the OxyBlot Protein Oxidation Detection kit (Merck, MA, USA) for immunoblot detection of carbonyl groups, according to the manufacturer's instructions. For cell experiments, the protein samples were solubilised by boiling in 1× Laemmli sample buffer containing 10% 2‐mercaptoethanol for 10 min. The samples were then loaded into 10% acrylamide gel for SDS‐PAGE and immune detection using the chemiluminescence method as previously described (Chan et al., 2013). Densitometry analysis was performed using the ImageLab software (Bio‐Rad Laboratories, RRID:SCR_008426).

### 2.7 Immunofluorescence and myotube diameter analyses

C2C12 myotubes grown on coverslips coated with Matrigel matrix basement membrane (Sigma‐Aldrich). At the end of the experiment, the myotubes were fixed in 4% paraformaldehyde in 1× PBS at room temperature for 30 min and the excess was quenched with 300 μM glycine. The fixed myotubes on coverslips were blocked and permeabilised in 10% bovine serum albumin, 2% Triton‐X in 1× PBS for 1 h at room temperature. The coverslips were then rinsed with 0.1% Tween 20 in 1× PBS and incubated overnight at 4°C with fluorophore conjugated antibodies against skeletal muscle myosin (F59 clone; AlexaFluor 488, Santa Cruz Biotechnology, USA, RRID:AB_670118). Unbound antibodies were removed by rinsing the coverslips in PBS with Tween 20 (PBST), excessive moisture was removed before mounting in Fluoroshield with DAPI (Sigma‐Aldrich, USA). Fluorescence images were captured using VS120 Olympus Virtual Slide Microscope (Olympus Life Science, Australia). The captured images were analysed using the Olympus cellSens software (Olympus Life Science, Australia). A minimum of 270 myotube diameters were measured for each condition.

### 2.8 Cell viability assay

The CellTiter 96 Aqueous One Solution (MTS) Cell Proliferation Assay (Promega, Australia) was used to determine cell viability of C2C12 myotubes, according to the manufacturer's instructions. Briefly, C2C12 myotubes stimulated with CSE or H_2_O_2_ were washed and incubated in media containing MTS reagent at 37°C and 5% CO_2_ for 1 h. Absorbance was then recorded at 490 nm using a plate reader (CLARIOstar Monochrome Microplate Reader; BMG Labtech, Australia). To validate the specificity of the assay, MTS reagent was added to unseeded culture plate containing CSE or H_2_O_2_ for 1 h before absorbance reading. No significant absorbance changes were observed in response to these stimuli verifying the reliability and specificity of the assay.

### 2.9 Enzyme‐linked immunosorbent assay

Mature IL‐6 and IGF‐1 released by the C2C12 myotubes were quantified using commercially available enzyme‐linked immunosorbent assay (ELISA) kits: murine IL‐6 ELISA Kit and murine IGF‐1 DuoSet ELISA Kit, according to the manufacturer's instructions. Briefly, plates were pre‐coated with capture antibody and then blocked with a universal diluent. Antibody standards were serially diluted in the universal diluent, constructing a 7‐point curve with a universal buffer as blank. Cell supernatant (undiluted) was then added in duplicates into the appropriate wells and agitated on a Thermomixer (Eppendorf, Germany) at 800 rpm for ≥2 h at room temperature. Wells were thoroughly washed with 0.05% Tween 20 in 1× PBS (PBST) before the detection antibody was added and agitated for 1 h at 800 rpm at room temperature. After washing, a developing solution with reporter enzyme and substrate was added and agitated for a further 1 h at room temperature. Absorbance was then recorded at 450 nm using a plate reader (CLARIOstar Monochrome Microplate Reader; BMG Labtech, Australia).

### 2.10 Data and statistical analysis

All data and statistical analysis comply with the recommendations on experimental design and analysis in pharmacology (Curtis et al., 2018). Data are presented as mean ± SEM unless otherwise stated. Statistical differences between treatments were determined by two‐tailed unpaired *t* test or analysis of variance (ANOVA) followed by Tukey's multiple comparison post hoc tests where appropriate. One‐way ANOVA were used for three or more unmatched groups. Two‐way ANOVA were used to analyse data when response was influenced by two independent factors of interest. All statistical analyses were performed using GraphPad PrismTM for Microsoft Windows® (Versions 8, Graphpad software®, USA, RRID:SCR_000306) where *P* < 0.05 was accepted as significant for all cases.

### 2.11 Materials

The suppliers of the materials used in these experiments are as follows: Winfield Red cigarettes (Phillip Morris, Australia); apocynin and DMSO (Sigma‐Aldrich, Australia); ketamine/xylazine and sodium pentobarbitone (Virbac Pty Ltd, Australia); acridine orange/ethidium bromide (Invitrogen, USA); Kwik‐Diff® reagent 1 fixative, High‐Capacity RNA‐to‐cDNA kit, pre‐developed TaqMan primers, cell culture reagents, Fluoromount‐G™, with DAPI, murine IL‐6 ELISA Kit, Pierce™ BCA Protein Assay Kit and SuperSignal™ West Femto Maximum Sensitivity Substrate for chemiluminescence detection (Thermo Fisher Scientific, USA);

RNeasy Mini Kit (Qiagen, Germany); C2C12 murine myoblasts (American Type Culture Collection, USA; CRL‐1772); H_2_O_2_ (Chem‐Supply, Australia); antibody for immunofluorescence (Santa Cruz Biotechnology, USA); MTS Cell Proliferation Assay (Promega, Australia); murine IGF‐1 DuoSet ELISA Kit (R&D Systems, USA); phosphorylation‐specific, actin antibodies and p62 for western blots (Cell Signaling Technology, USA); all other antibodies for western blots (Abcam, USA).

### 2.12 Nomenclature of targets and ligands

Key protein targets and ligands in this article are hyperlinked to corresponding entries in the IUPHAR/BPS Guide to PHARMACOLOGY (http://www.guidetopharmacology.org) and are permanently archived in the Concise Guide to PHARMACOLOGY 2019/20 (Alexander, Christopoulos et al., 2019; Alexander, Fabbro et al., 2019).

## 3 RESULTS

### 3.1 Apocynin treatment attenuates the pro‐inflammatory lung response induced by CS exposure

**FIGURE 1:**
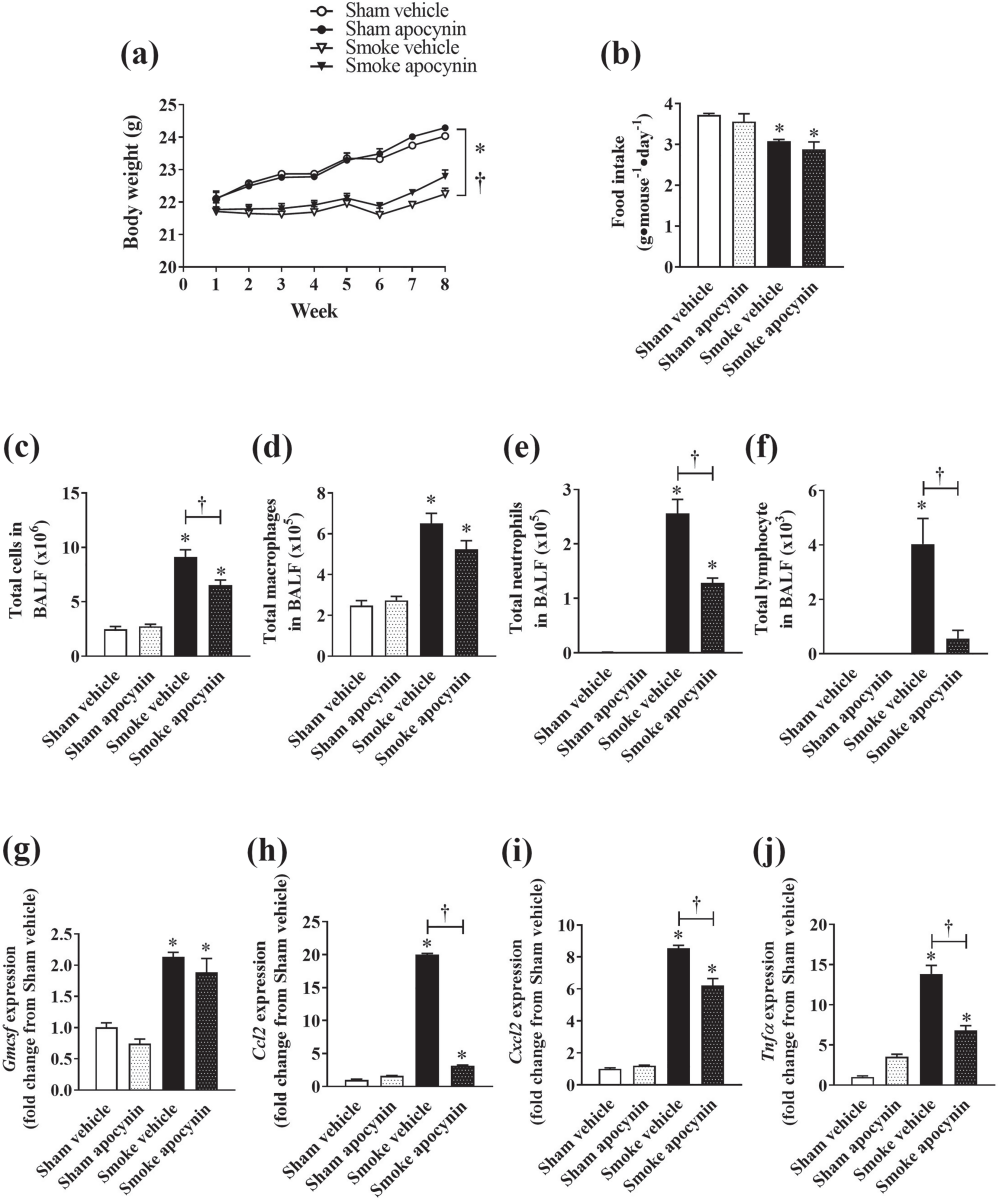
Effect of apocynin on body weight, food intake and lung inflammation induced by CS exposure. Mice were exposed to CS (smoke) or room air (sham) for 8 weeks with or without i.p. injection of apocynin (5 mg·kg^−1^·day^−1^) or vehicle (saline). Progressive body weight of CS‐exposed (smoke) and room air‐exposed (sham mice with or without apocynin (a) and average food intake (b) across the experimental period. Total number of cells (c), macrophage (d), neutrophils (e) and lymphocytes (f) in BALF. Quantitative PCR was performed to assess the expression of *Gmcsf* (g), *Ccl2* (h), *Cxcl2* (i), and *Tnfα* (j) in homogenised lung tissues. Data are expressed as mean ± SEM (n = 8–10 mice per group) from one of two independent experiments and analysed by two‐way ANOVA with multiple comparisons and Tukey post hoc test. *P < 0.05, significantly different from the relevant sham group; †*P <* 0.05, significantly different as indicated

Mice displayed no significant difference in starting body weight and food intake. However, CS exposure concomitantly reduced body weight gain (~7% loss) and food intake (~17% loss) which were unaffected by apocynin (5 mg kg^‐1^) administration, suggesting apocynin did not affect growth or appetite of these mice at the administered dosage (Figure 1a,b). In line with the reduced body weight gain, tissue mass of testicular (30%) and retroperitoneal (38%) white adipose tissue (WAT), heart (9%) and spleen (21%) were also reduced by CS exposure. However, all these changes, except those for the heart, were prevented by apocynin treatment (Table 2). To examine whether apocynin treatment was effective in attenuating the direct impact of CS on immune cell recruitment to the lung, we performed differential cell count analyses on the bronchoalveolar lavage fluid (BALF). CS exposure caused a 3.7‐fold increase in total cell infiltration which was attributed to a marked increase in the number of macrophages, neutrophils and lymphocytes (Figure 1c–f). In line with this, CS exposure caused a marked increase in gross lung weight (Table 2) and the expression of key pro‐inflammatory cytokines/chemokines in the lungs, including granulocyte–macrophage colony‐stimulating factor (Gmcsf), CCL2 (Ccl2), CXCL2 (*Cxcl2*) and TNF‐α (Tnfα *)* (Figure 1g–j). Apocynin treatment significantly attenuated the CS‐induced cell content of BALF as shown by a 28% reduction in total cell counts (Figure 1c), 50% reduction in neutrophil counts (Figure 1e) and 86% reduction in lymphocyte counts in the CS‐exposed mice (Figure 1f), without significant alterations in macrophage counts (Figure 1d). Accordingly, the CS‐induced expression of *Ccl2*, *Cxcl2*, *Tnfα* in the lungs were significantly attenuated by 84% (Figure 1h), 27% (Figure 1i) and 51% (Figure 1j), respectively; whereas the expression of *Gmcsf* remained elevated despite apocynin treatment (Figure 1g). This attenuation of pro‐inflammatory factors in the lung by apocynin appeared to be specific to the CS exposure, as no significant effects in BALF cellularity (Figure 1c–f) and gene expression (Figure 1g–j) were observed in the sham‐exposed mice.

**TABLE 2:** Summary of tissue weights from sham and CS‐exposed mice, with or without apocynin treatment.

Tissue	Sham vehicle	Sham apocynin	Smoke vehicle	Smoke apocynin
Body weight (g)	25.23 ± 0.49	25.20 ± 0.58	22.25 ± 0.63*	22.98 ± 0.53*
Soleus (mg)	7.32 ± 0.77	7.66 ± 0.87	6.76 ± 0.80*	7.16 ± 0.65
Gastrocnemius (mg)	122.9 ± 3.4	124.1 ± 2.4	116.4 ± 2.6	118.9 ± 2.5
Plantaris (mg)	15.79 ± 0.5	16.2 ± 0.4	15.3 ± 0.3	15.0 ± 0.4
Testicular WAT (mg)	595.9 ± 52	533.6 ± 66	419.6 ± 46*	513.9 ± 41
Retroperitoneal WAT (mg)	106.5 ± 8.0	100.9 ± 15	66.15 ± 7.2*	89.0 ± 10
Lung (mg)	227.9 ± 8.0	247.1 ± 6.3	281.9 ± 17.9*	296.3 ± 16.0*
Heart (mg)	125.8 ± 3.1	126.9 ± 3.1	114.6 ± 4.6*	115.4 ± 3.8*
Spleen (mg)	88.8 ± 3.2	95.68 ± 2.1	69.8 ± 6.6*	74.39 ± 2.0
Liver (mg)	1312 ± 78	1103 ± 40	1079 ± 37	982.7 ± 41
Kidney (mg)	369.3 ± 12.4	372.1 ± 8.0	350.1 ± 14.1	340.7 ± 15.7

*Note:* Data are expressed as mean ± SEM.

* *P <* 0.05 significantly different from the corresponding Sham; two‐way ANOVA with multiple comparisons and Tukey post hoc test.

### 3.2 Apocynin treatment prevents loss of skeletal muscle function caused by CS exposure

**FIGURE 2:**
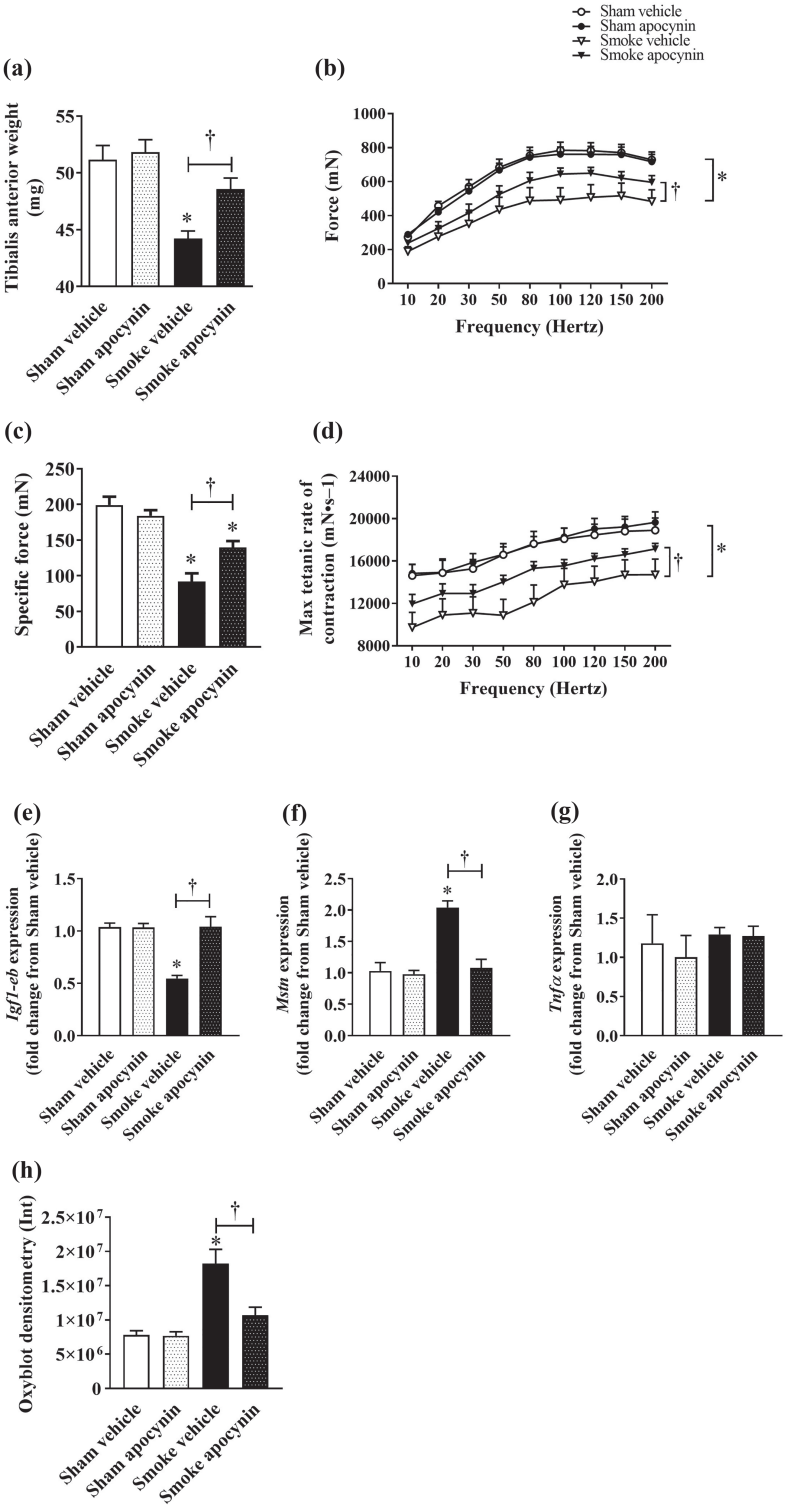
Effect of CS exposure on tibialis anterior (TA) muscle weight, contractile performance and homeostatic changes. TA muscle weight (a), maximum contractile force (b), specific force at 120 Hz (c), and maximum contraction rate measurements (d) were analysed at the end of the experimental period. Quantitative PCR was performed to assess the expression of *Igf1‐eb* (e), *Myostatin* (f) and *Tnfα* (g) in homogenised TA muscle. Densitometry analysis of oxyblots for total oxidized proteins in the TA muscle (h). Data are expressed as mean ± SEM (n = 8–10 mice per group, Oxyblot analysis was conducted on n = 5 mice per group) from one of two independent experiments and analysed by two‐way ANOVA with multiple comparisons and Tukey post hoc test. *P < 0.05, significantly different from the relevant sham group; †*P <* 0.05, significantly different as indicated

Eight weeks of CS exposure resulted in a loss of skeletal muscle mass in mice similar to that observed in human smokers, shown as a 10% reduction in gross weight of the tibialis anterior (TA) muscle (Figure 2a) which is a prime mover of the hind limb, predominated by fast‐twitch myofibres. CS exposure also caused an ~8% reduction in the weight of soleus (Table 2) which is a predominantly slow‐twitch fibre muscle of the hind limbs (Timson et al., 1985), suggesting the muscle wasting effect of CS exposure was unrelated to the fibre composition, in our model. In addition to the loss of muscle mass, CS exposure also resulted in a significant reduction in contractile force (Figure 2b) and maximum contraction rate (Figure 2d) of the TA muscles which translated to a 54% decrease in specific force generated (Figure 2c), suggesting CS exposure caused muscle weakness. In addition to preventing the loss of TA mass, apocynin treatment attenuated the CS‐induced skeletal muscle weakness as shown by the improved contractile force, maximum contraction rate and specific force (Figure 2a–d).

We next conducted quantitative PCR (qPCR) analyses to examine the molecular changes within the TA muscles. In line with the loss of mass and function, CS exposure resulted in a 50% reduction in *Igf1‐eb* (a precursor isoform of muscle‐derived IGF‐1; Figure 2e) and a twofold increase in *Mstn* (myostatin; Figure 2f) expression which were completely prevented by apocynin treatment. *Tnfα* expression remained unaltered regardless of CS exposure or apocynin treatment, suggesting the CS‐induced phenotypical and molecular changes are unlikely to involve myocellular inflammation. Lastly, our oxyblot analysis revealed a 2.3‐fold increase in protein carbonylation of TA muscles following CS exposure which was completely prevented by apocynin treatment (Figure 2 H), suggesting the protective effects of apocynin in vivo may be related to its ability to antagonise the oxidative burden evoked by CS exposure.

### 3.3 Exposure to oxidative insult (H_2_O_2_) or CSE results in a similar degree of wasting in C2C12 myotubes without cellular inflammation

**FIGURE 3:**
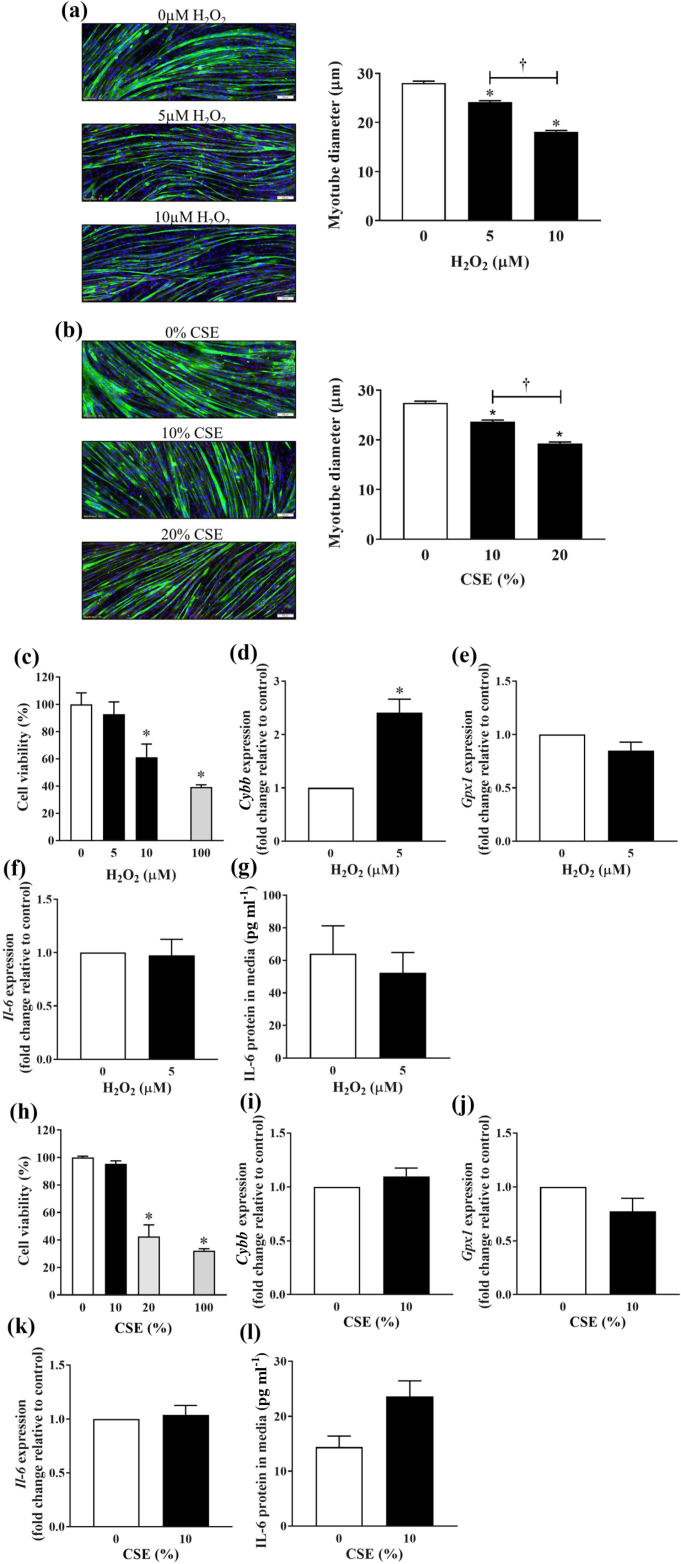
Effect of H_2_O_2_ exposure and CSE exposure on C2C12 myotube size, viability and cellular stress response. C2C12 myotubes were exposed to increasing concentrations of either H_2_O_2_ (a) or CSE (b) for 24 h. Cell viability was assessed using the MTS assay following H_2_O_2_ exposure (c) or CSE exposure (h). Quantitative PCR was performed to assess the expression of *Cybb* (d and i), *Gpx1* (e and j) and *Il‐6* (f and k). IL‐6 released into the medium in response to H_2_O_2_ (g) or CSE (l) was quantified using ELISA. For myotube size assessments, data are represented as mean ± SEM of three independent experiments (n = 270 myotubes counted per condition); other data are represented as mean ± SEM of three independent experiments (n = 7–9 per condition). *P < 0.05, significantly different from the relevant sham group; †*P <* 0.05, significantly different as indicated. Scale bars = 100 μm (a and b)

To further dissect the importance of oxidative stress on muscle dysfunction, we compared the direct effects of a ROS, H_2_O_2_ and of CSE on C2C12 myotubes. Both H_2_O_2_ and CSE exposure resulted in a similar dose‐dependent reduction in myotube size (Figure 3a,b). Unlike that of the higher dose, the reduction in myotube size induced by low doses of H_2_O_2_ (5 μM) or CSE (10%) did not affect cell viability (Figure 3c,h). In line with the presence of oxidative stress, exposure to H_2_O_2_ (5 μM) elicited a robust expression of *Cybb* which is involved in the formation of ROS in muscle (Figure 3d) (Sakellariou et al., 2013). Unlike that of H_2_O_2_, no significant induction of *Cybb* expression was observed after exposure to 10% CSE (Figure 3i). Meanwhile, the expression of glutathione peroxidase 1 (*Gpx1*), a detoxifying enzyme that scavenges H_2_O_2_, was unaltered by either of the stimuli (Figure 3e,j). Noteworthy, we observed no cellular inflammatory response as shown by the *Il‐6* gene expression (Figure 3f,k) and release of IL‐6 (Figure 3g) at sub‐lethal concentrations, although a strong trend (*P* = 0.06) of IL‐6 release was observed under 10% CSE (Figure 3l). This suggests the oxidative stress‐driven myofibre wasting can occur without any detectable cellular inflammation, replicating that of our in vivo model.

### 3.4 CSE‐driven myofibre wasting suppresses myogenic factor production without affecting atrophy related genes

**FIGURE 4:**
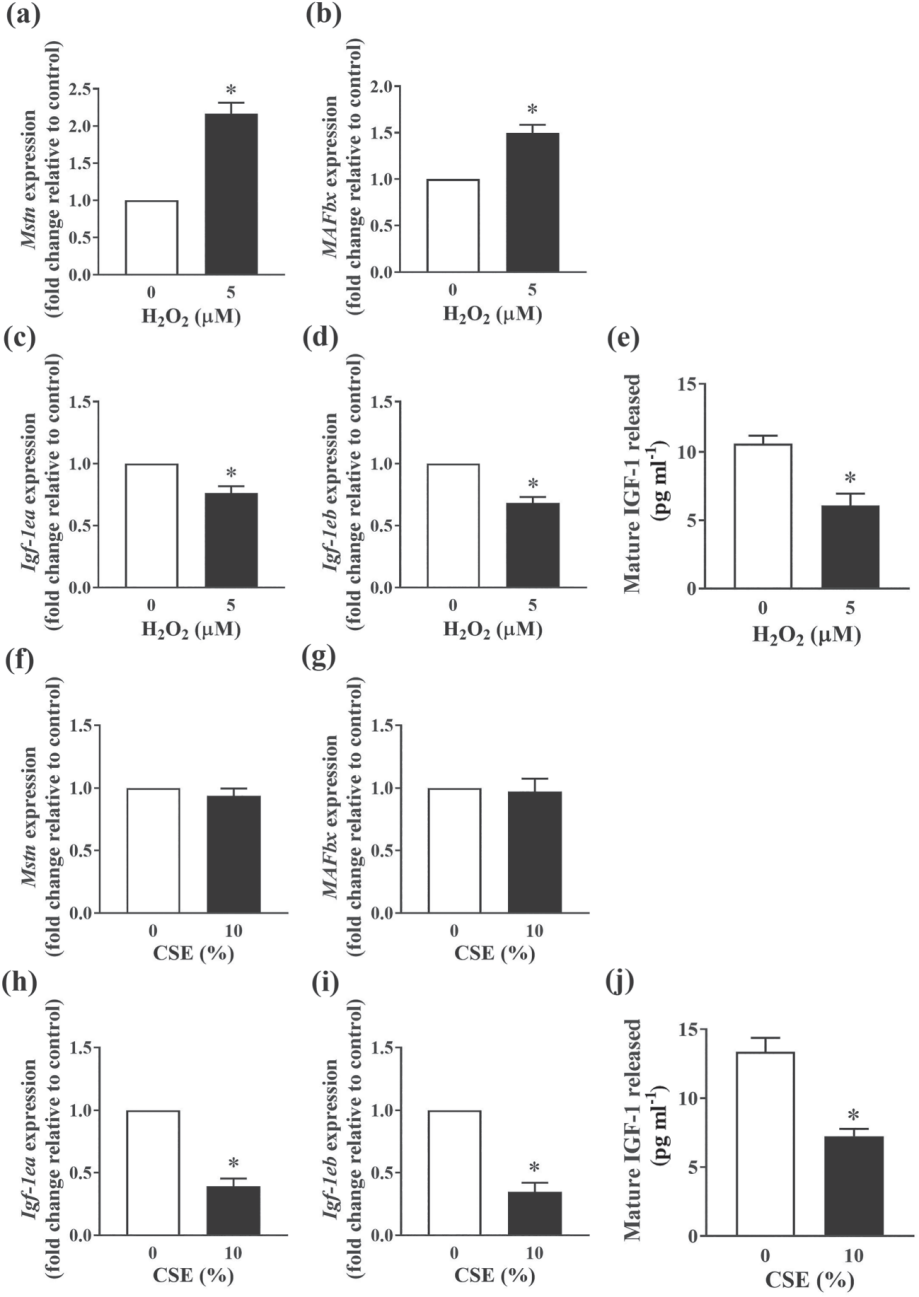
Effect of H_2_O_2_ exposure and CSE exposure on proteostasis in C2C12 myotubes. C2C12 myotubes were exposed to sub‐lethal concentrations of either H_2_O_2_ or CSE for 24 h. Quantitative PCR was performed to assess the expression of *Mstn* (a and f), *MAFbx* (b and g), *Igf‐1ea* (c and h) and *Igf‐1eb* (d and i). Mature IGF‐1 released into the medium in response to H_2_O_2_ (e) or CSE (j) was quantified using ELISA. Data are represented as mean ± SEM of three independent experiments (n = 7–9 per condition). *P < 0.05, significantly different from control (i.e., concentration 0)

In humans, smoking has been demonstrated to inhibit muscle protein synthesis and increase the expression of genes associated with defective muscle maintenance such as *Mstn* and muscle atrophy F‐box (*MAFbx)* (Petersen et al., 2007). Indeed, direct exposure of myotubes to sub‐lethal concentrations of H_2_O_2_ also resulted in a significant induction of both *Mstn* (Figure 4a) and *MAFbx* (Figure 4b), whereas the production and release of IGF‐1, a potent driver of protein synthesis and myogenesis (Florini et al., 1996), were concomitantly suppressed (Figure 4c–e). As observed with H_2_O_2_, direct exposure to CSE also suppressed the production and release of IGF‐1 (Figure 4h–j). However, the expression of *Mstn* (Figure 4f) and *MAFbx* (Figure 4g) remained largely unaltered, suggesting the deleterious effects of CSE exposure on myofibre wasting may predominantly lie in the suppression of IGF‐1 mediated protein synthesis.

### 3.5 Apocynin prevents the suppression of myogenic factor expression and myofibre wasting induced by CSE and H_2_O_2_

**FIGURE 5:**
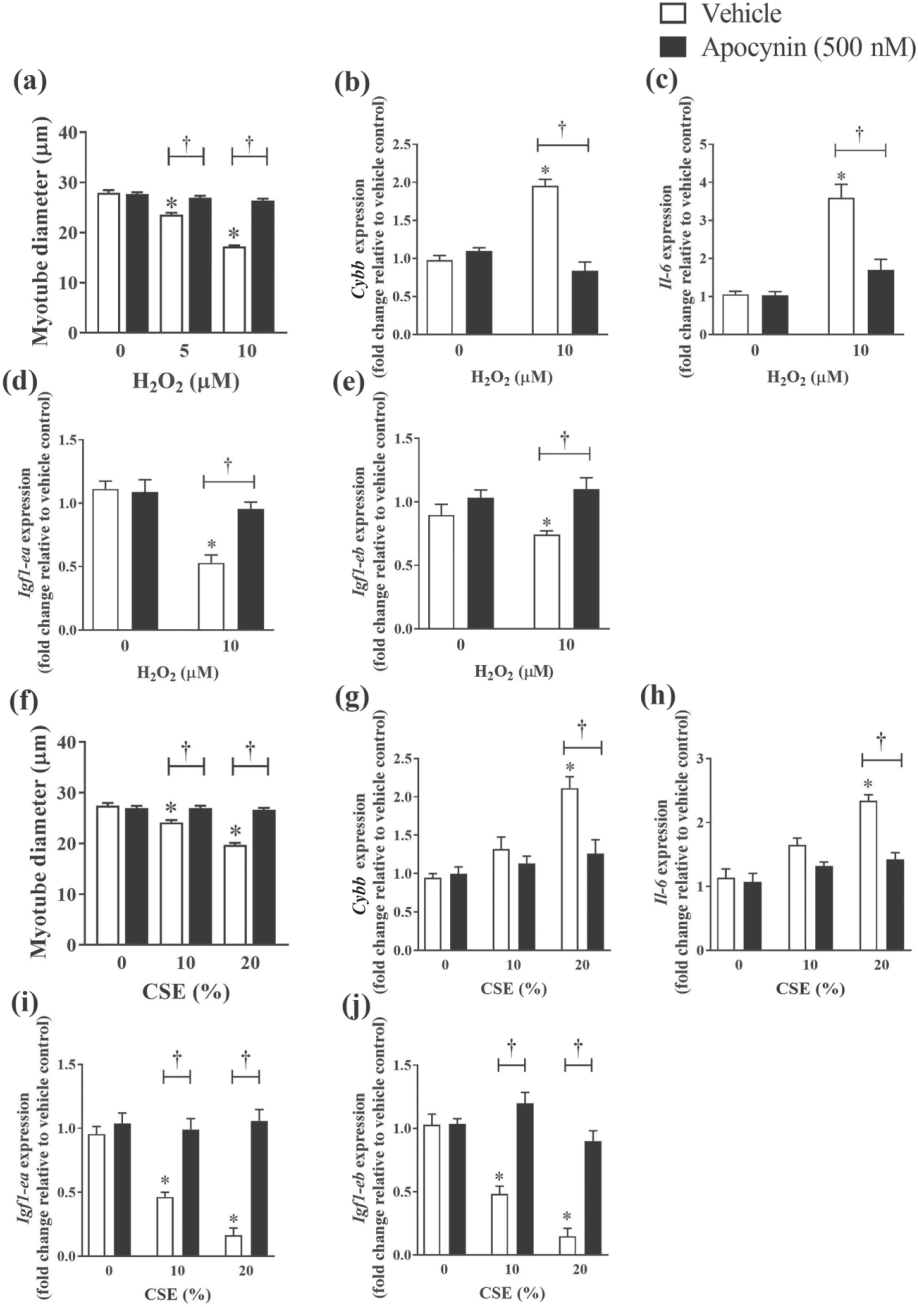
Effect of apocynin on C2C12 myotubes size and cellular stress. C2C12 myotubes were exposed to increasing concentrations of either H_2_O_2_ or CSE with or without apocynin (500 nM) for 24 h. Changes in myotube diameters were quantified (a and f) from three independent experiments (n = 270 myotubes counted per condition). Quantitative PCR was performed to assess the expression of *Cybb* (b and g), *Il‐6* (c and h), *Igf1‐ea* (d and i) and *Igf1‐eb* (e and j). Data are represented as mean ± SEM of three independent experiments (n = 7–9 per condition unless otherwise stated). *P < 0.05, significantly different from vehicle control (i.e., concentration 0); †*P <* 0.05, significantly different as indicated

Under unstimulated conditions, apocynin treatment had no effect on myotube diameters (Figure 5a,f). However, treatment with apocynin prevented myofibre wasting (i.e., reduction in myofibre size) elicited by different concentrations of H_2_O_2_ (Figure 5a) or CSE (Figure 5f). Apocynin treatment completely attenuated the up‐regulated expression of *Cybb* (Figure 5b,g) and *Il‐6* (Figure 5c,h) driven by H_2_O_2_ or CSE. Moreover, apocynin normalised the expression of *Igf1‐ea and Igf1‐eb*, suggesting apocynin was able to antagonise the oxidant‐dependent and oxidant‐independent effects of CSE on myofibre wasting.

### 3.6 The protective effects of apocynin are attributable to preserved proteostatic signalling

**FIGURE 6:**
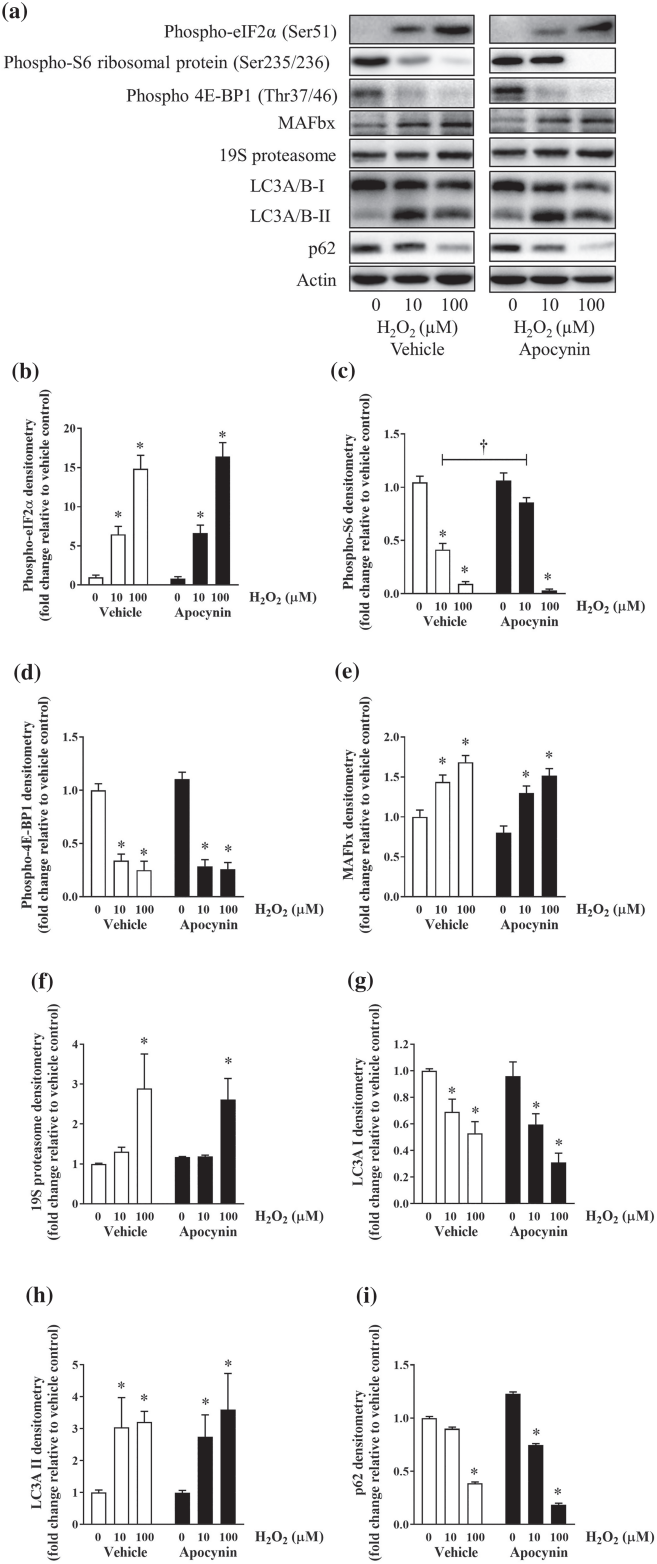
Effect of H_2_O_2_ on proteostasis in C2C12 myotubes. C2C12 myotubes were exposed to increasing concentrations of H_2_O_2_ with or without apocynin (500 nM) for 24 h. At the end of the experiment, samples were collected for western blotting analysis. Representative images of the western blots (a) and their respective densitometry analyses (b–i). Data are represented as mean ± SEM of three independent experiments (n = 6 per condition). *P < 0.05, significantly different from vehicle control (i.e., concentration 0); †*P <* 0.05, significantly different as indicated

The main function of muscle‐derived IGF‐1 is to promote protein synthesis and muscle growth via the action of an intracellular signal transducer, mTOR (Nicklin et al., 2009). Given the expression of muscle‐derived IGF‐1 was suppressed by CS (Figure 2e) and CSE (Figure 4c–e,h–j) exposure, it was possible that the key signal transduction pathways responsible for maintaining balance between protein synthesis and breakdown (i.e., proteostatic signalling) were affected. H_2_O_2_ exposure concentration‐dependently decreased the phosphorylation level of S6 ribosomal protein and eukaryotic translation initiation factor 4E‐binding protein 1 (4E‐BP1; Figure 6a,c,d), which are the key downstream effectors of mTOR (Schiaffino & Mammucari, 2011). The phosphorylation status of a key repressor of protein synthesis, eukaryotic translation initiation factor 2A (eIF2α; Figure 6a,b), was increased by 5 to 15‐fold, suggesting a global inhibition of protein synthesis. In line with the mRNA expression (Figure 4a), H_2_O_2_ increased the protein abundance of MAFbx (~50%), a muscle‐specific E3 ubiquitin ligase (Figure 6a,e). Furthermore, a significant increase in abundance of the 19S proteasome (S5a), a regulatory subunit of the 26S proteasomal complex, was observed following exposure to 100 μM of H_2_O_2_ (Figure 6a,f), suggesting the activation of the ubiquitin‐proteasome system (UPS). H_2_O_2_ exposure also resulted in the activation of autophagic pathway shown by the conversion of LC3A/B‐I to LC3A/B‐II (Figure 6a,g,h) and decreased p62 abundance (Figure 6a,i). Apocynin treatment maintained phosphorylated S6 ribosomal protein expression against 10 μM of H_2_O_2_ but not 4E‐BP1 or eIF2α (Figure 6a–d). Meanwhile, no significant effects were detected for the activation of UPS and autophagic pathways (Figure 6a,e–i) suggesting the protective effects of apocynin are unlikely to be modulated through protein degradative pathways.

**FIGURE 7:**
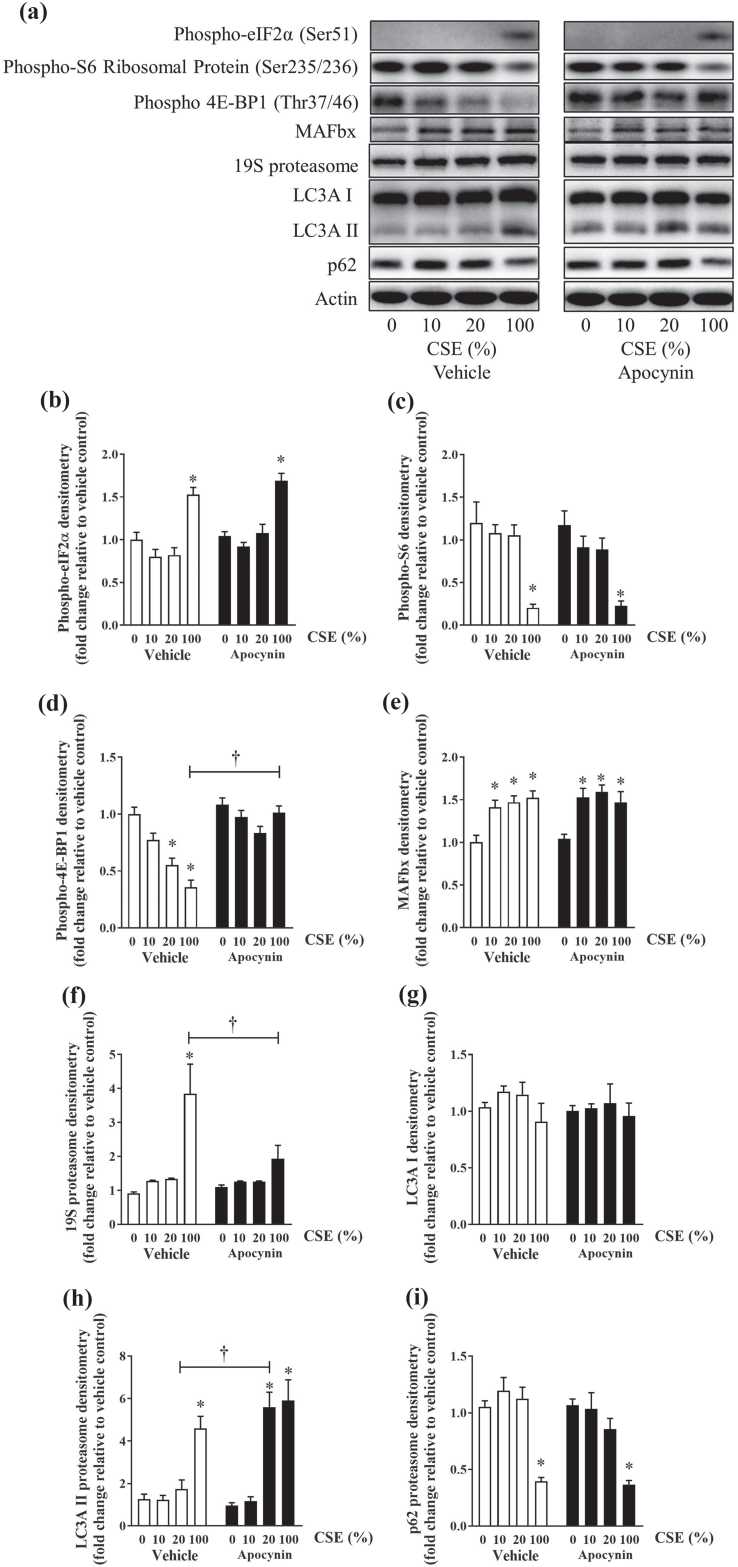
Effect of CSE on proteostasis in C2C12 myotubes. C2C12 myotubes were exposed to increasing concentrations of CSE with or without apocynin (500 nM) for 24 h. At the end of the experiment, samples were harvested for western blotting analysis. Representative images of the western blots (a) and their respective densitometry analyses (b–i). Data are represented as mean ± SEM of three independent experiments (n = 6 per condition). *P < 0.05, significantly different from vehicle control (i.e., concentration 0); †*P <* 0.05, significantly different as indicated

Meanwhile, exposure of myotubes to submaximal concentrations of CSE did not evoke the phosphorylation of eIF2α (Figure 7a,b) or decrease the phosphorylated S6 ribosomal protein expression (Figure 7a,c), although a concentration‐dependent reduction in the phosphorylation levels of 4E‐BP1 was observed (Figure 7a,d). As found with H_2_O_2_, CSE exposure increased the abundance of MAFbx (Figure 7a,e), although no detectable changes in 19S proteasome protein were observed until the maximal concentration (100%) of CSE was used (Figure 7a,f). Likewise, exposure to the maximal concentration of CSE resulted in the activation of autophagic pathway shown as the LC3A/B‐I to LC3A/B‐II conversion (Figure 7a,g,h) and decrease in p62 abundance (Figure 7a,i), but no significant effects were observed under submaximal conditions (10–20% of CSE). Apocynin treatment preserved the phosphorylation of 4E‐BP1 without affecting that of eIF2α or S6 ribosomal protein (Figure 7a,b–d). Apocynin treatment completely blocked the enrichment of 19S proteasome elicited by maximal concentration of CSE (Figure 7a,f). To our surprise, the conversion of LC3A/B‐I to LC3A/B‐II which was undetectable at submaximal CSE concentrations under vehicle condition, became apparent starting at 20% CSE concentration, suggesting apocynin may selectively enhance cellular autophagic responses in the CSE‐exposed myotubes.

## 4 DISCUSSION

The present study found that apocynin treatment was effective in attenuating lung inflammation and prevented the skeletal muscle dysfunction resulting from CS exposure. Our molecular analysis found that the CS‐induced muscle dysfunction is attributed to oxidative stress and impaired muscle‐derived IGF‐1 expression which leads to a disruption of proteostatic signalling. Apocynin effectively modulated oxidative stress, thereby preserving muscle‐derived IGF‐1 expression and the downstream proteostatic signalling in myofibres, protecting them from the damaging effects of CS/CSE exposure.

In the lungs, CS exposure elicited an abnormal inflammatory response, which may promote mucous metaplasia and lung destruction leading to the manifestation of chronic bronchitis and emphysema (O'Donnell et al., 2006). Neutrophils have been suggested to be a key driver of these deleterious effects in the lungs, by secreting a number of proteases, such as matrix metalloproteinases and neutrophil elastases (Vlahos et al., 2006). These proteases degrade components of the pulmonary extracellular matrix leading to the destruction of the lung parenchyma (Vlahos et al., 2006). Meanwhile, neutrophilic proteases may perpetuate lung inflammation by acting on proteinase‐activated receptors (PARs)(Jenkins et al., 2006; Scotton et al., 2009). Destruction of the lung parenchyma and persistent inflammation not only drives the development of airflow limitation and emphysema, but also compromises the integrity of epithelial lining of the airway (Vlahos et al., 2006). This increases lung permeability allowing for the overspill of pro‐inflammatory mediators into the systemic circulation, which has been postulated to be a key mechanism for the onset of skeletal muscle dysfunction (Bernardo et al., 2015; Passey et al., 2016).

Indeed, skeletal muscle dysfunction was observed following 8 weeks of CS exposure, characterised by the loss of mass and contractile function (Figure 2a–d). In patients with COPD, muscle dysfunction is most frequently reported in the lower limbs than the upper limbs (Gea et al., 2001; Man et al., 2003), suggesting that leg muscles are more susceptible to dysfunction in patients with COPD. Strikingly, symptoms of muscle weakness, which are hallmarks of functional impairment, have been reported in smokers without detectable decline in respiratory function (Maltais et al., 2014). This not only suggests that CS may directly impair leg muscle function, but also that the onset of limb muscle dysfunction may well precede that of respiratory symptoms. On this note, impaired quadriceps function was detected in asymptomatic smokers with matching physical activity levels to non‐smokers, which may be attributed to an acute toxicity of CS exposure on oxygen delivery and mitochondrial function (Wust et al., 2008).

In addition to exerting acute toxicity, our study suggests that muscle loss and dysfunction may also arise from chronic oxidative stress elicited by repeated CS exposure. It is understood that CS represents an external source of oxidants (>10^16^ free radicals per puff) which exert adverse effects on tissues through oxidative damage of biological structures (Bartalis et al., 2007). Moreover, CS also activates inflammatory cells of the airway and lungs which may enhance oxidant production in pulmonary and extra‐pulmonary tissues. Through these sources, chronic CS exposure generates transient and repeated bouts of oxidative stress which may modify key proteins involved in muscle metabolism or function, leading to the manifestation of muscle dysfunction seen in patients with COPD (Barreiro et al., 2010). Indeed, our results demonstrated the presence of oxidative stress and increased protein oxidation following CS exposure. This took place independent of muscle inflammation but was linked to an altered myogenic homeostasis characterised by a blunted expression of IGF‐1 and increased expression of myostatin, suggesting a disrupted proteostasis. In C2C12 myotubes, we found that oxidative stress suppressed mTOR‐driven protein synthesis, while activating the UPS degradative pathway resulting in myofibre wasting. Myostatin is a member of the TGF‐β family and a potent inducer of muscle atrophy. By inhibiting myogenic signalling, myostatin activates the UPS pathway through Forkhead box class O 3a (FoxO3a), thereby promoting the expression of the muscle‐specific ubiquitin ligases: Muscle RING finger 1 (MuRF1) and MAFbx, resulting in a net loss of muscle protein and atrophy (Zhou et al., 2010). In muscle, Sriram et al (Sriram et al., 2011) demonstrated that oxidative stress is a potent stimulator of myostatin expression. Intriguingly, the same study also showed that myostatin itself also causes oxidative stress via the action of the transcription factor NFκB and Nox2, meaning that a self‐perpetuated mechanism may exist to sustain protein degradation in atrophic muscles. Nevertheless, these findings highlight the instrumental role of oxidative stress in CS‐induced myostatin expression and muscle loss observed in our study.

In accordance with this, attenuation of oxidative stress by apocynin markedly ameliorated the CS‐induced lung inflammation and muscle dysfunction. In the muscle, apocynin prevented the induction of myostatin and its inhibitory effects on myogenic signalling, thereby preserving muscle proteostasis. In human COPD patients, muscle loss has been postulated to be a result of unintended weight loss due to malnutrition (Collins et al., 2019). Our in vivo data certainly reflects an association between loss of TA mass with reduced weight gain and food intake by CS exposure. However, apocynin treatment was able to preserve muscle mass and function despite both weight gain and food intake were suppressed, suggesting the CS‐induced muscle loss is unlikely a result of simple weight loss from malnutrition. Moreover, loss of muscle mass was mainly observed in the TA and soleus muscles, but not the gastrocnemius and plantaris, highlighting the selective nature of CS‐induced muscle loss. Although malnutrition and weight loss may be a major contributor to muscle loss in advanced COPD where respiratory function is severely compromised, they are unlikely to account for the direct effects of CS‐induced muscle loss, observed in this study.

Another interesting finding of the present study is that the impaired contractile function by CS exposure was only partly improved by apocynin, despite a fully preserved muscle mass. This apparent mismatch raises an important notion that muscle mass and function may not always correlate in a linear fashion in patients with COPD, unlike that in healthy individuals. In agreement with this, Mantoani et al. (2017) reported no correlations between muscle mass and function assessed by quadriceps maximal voluntary contraction, although baseline physical activity was found to be related to greater muscle strength. In addition to its deleterious effects on muscle mass, CS exposure has been shown to directly impair excitation–contraction coupling (Nogueira et al., 2018) suggesting the contractile apparatus in muscle is sensitive to redox modifications. Barreiro et al. (2010) reported that a number of muscle proteins involved in force generation are subjected to post‐translational oxidative modifications, including ATP synthase and actin. Oxidative modifications of protein, such as carbonylation, may result in loss of protein function and accelerated degradation by the UPS (Barreiro et al., 2010) which may offer an explanation for the impaired contractile function observed in our study. Collectively, these findings suggest that the relationship between muscle mass and function is unlikely to be linear, particularly in smokers or patients with COPD. Further studies should be mindful of factors that may influence this relationship, such as muscle of interest, the type of assessment chosen, age, sex and disease severity of the test subject, when designing interventional trials for COPD patients aiming to examine muscle changes.

As muscle mass and function may not be correlated, in the context of COPD, the finding that not all leg muscles display susceptibility to CS‐induced muscle loss would prompt a new set of research questions on (1) whether strength is preserved in muscles that are seemingly unaffected by mass loss and (2) what effect does apocynin have on the contractile function of these muscles? Due to the limitation of the present study, we are unable to shed further light on these questions. Meanwhile, the different outcomes of Nox‐related oxidative stress in the lung (heightened inflammation), compared with that in skeletal muscle of the hind limb (loss of mass and function without inflammation), certainly imply that Nox activation does not always evoke cellular inflammation. The onset of this type of cellular inflammation appears to be dependent on several factors including the antioxidant status of the tissue, resident immune cells, mode and duration of stress exposure (Bernardo et al., 2015; Singel & Segal, 2016). Another point to note is that our in vivo data was derived in male mice, and male and female mice have been demonstrated to respond differently to cigarette smoking (Tam et al., 2016). Hence, future studies in female mice are needed to address this knowledge gap. Regarding apocynin, it seems to act as a prodrug, which must be initially oxidised into its dimeric form, diapocynin, in order to be active (Johnson et al., 2002). Supporting this, Ximenes et al. (Ximenes et al., 2007) reported the isolation of diapocynin in apocynin‐treated neutrophils, and that the purified forms of diapocynin have been suggested to be more effective than apocynin itself (Kanegae et al., 2010; Mora‐Pale et al., 2009). However, we did not test the effectiveness of diapocynin to make a valid comparison in the present study. Despite the controversies regarding its potency and selectivity as a Nox inhibitor, apocynin remains one of the most promising drugs for experimental models of disease involving ROS since its characterisation in 1994.

In summary, we show that Nox‐driven oxidative stress may be an underlying mechanism for the skeletal muscle loss and dysfunction caused by CS exposure. The induction of oxidative stress disrupts proteostasis by dampening myogenic signalling and enhancing UPS activation, resulting in muscle loss. Meanwhile, the oxidative modification of muscle proteins may also give rise to contractile impairment. By inhibiting Nox‐driven oxidative stress, apocynin treatment attenuated lung inflammation and preserved myofibrillar proteostasis, thereby preventing muscle loss and dysfunction. Therefore, targeted inhibition of oxidative stress may be utilised to improve pulmonary and systemic outcomes associated with COPD.

## AUTHOR CONTRIBUTIONS

Concept and design: R.V., S.M.H.C., I.B; acquisition of data: I.B., C.M., S.M.H.C., H.J.S; data analysis and interpretation: S.M.H.C., I.B., C.M., S.N.D.L., H.J.S., A.D., K.B., K.M., S.S., S.B., R.V.; technical assistance: K.M., A.D., K.B.; drafting, editing, and/or critical revision of the manuscript for intellectual content: all authors; R.V. also provided the resources for the work to be performed and is the senior investigator ensuring accuracy and integrity of the work.

## DECLARATION OF TRANSPARENCY AND SCIENTIFIC RIGOUR

This Declaration acknowledges that this paper adheres to the principles for transparent reporting and scientific rigour of preclinical research as stated in the BJP guidelines for Design & Analysis, Immunoblotting and Immunochemistry, and Animal Experimentation, and as recommended by funding agencies, publishers and other organisations engaged with supporting research.

## CONFLICT OF INTEREST

The authors declare no conflicts of interest.

## Supporting information

**Data S1.** Supporting information

## Data Availability

The data that support the findings of this study are available from the corresponding author upon reasonable request. Some data may not be made available because of privacy or ethical restrictions.
