# Supplementary material for: Apocynin prevents cigarette smoking‐induced loss of skeletal muscle mass and function in mice by preserving proteostatic signalling
Source: Br J Pharmacol. 2021 Jun 8;178(15):3049–66. doi: 10.1111/bph.15482 (PMC8362135; doi:10.1111/bph.15482)

Figure S1 (Oxyblots for Figure 2)

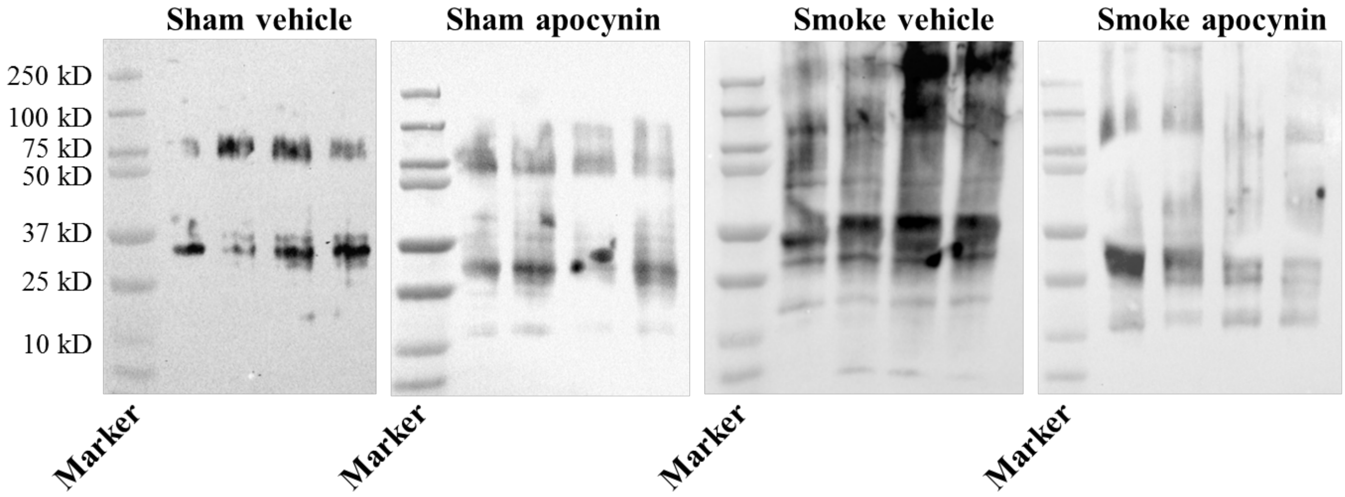

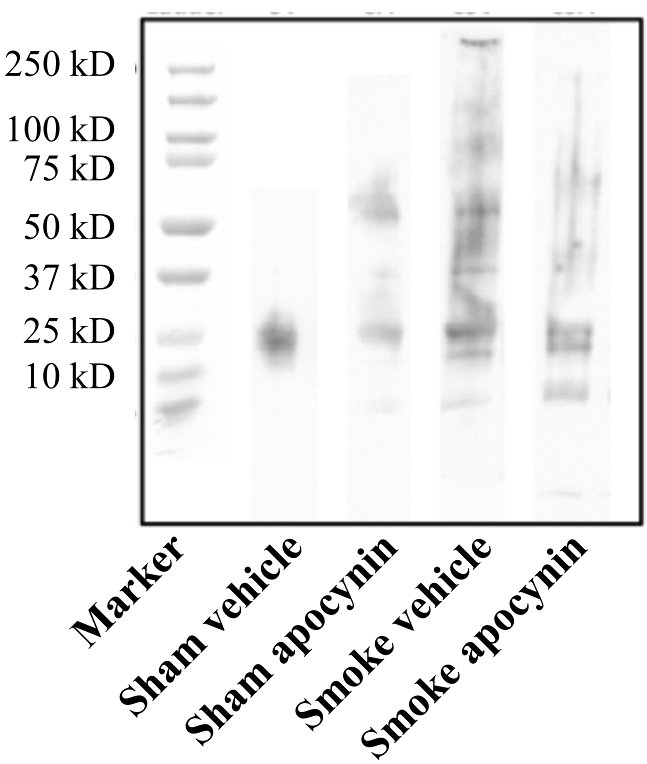

Each lane represents sample from 1 mouse

Figure S2

Phospho-eIF2α (Ser51)

38 kDa

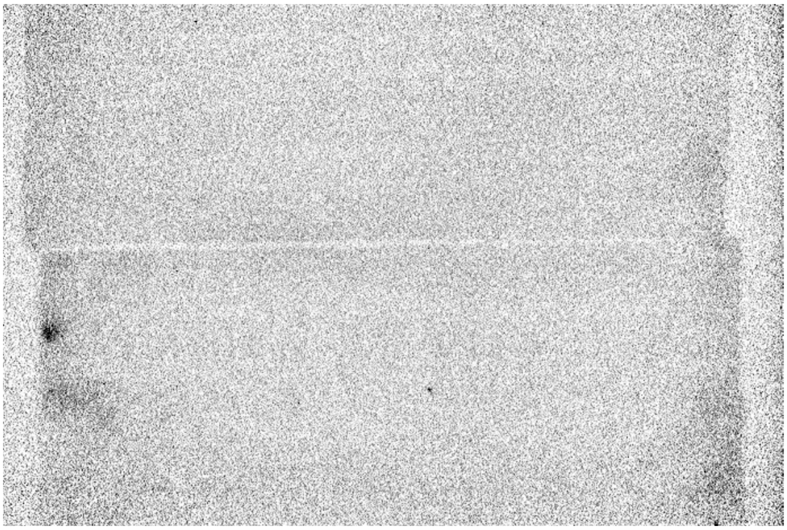

Phospho-S6 ribosomal protein (Ser235/236)

32 kDa

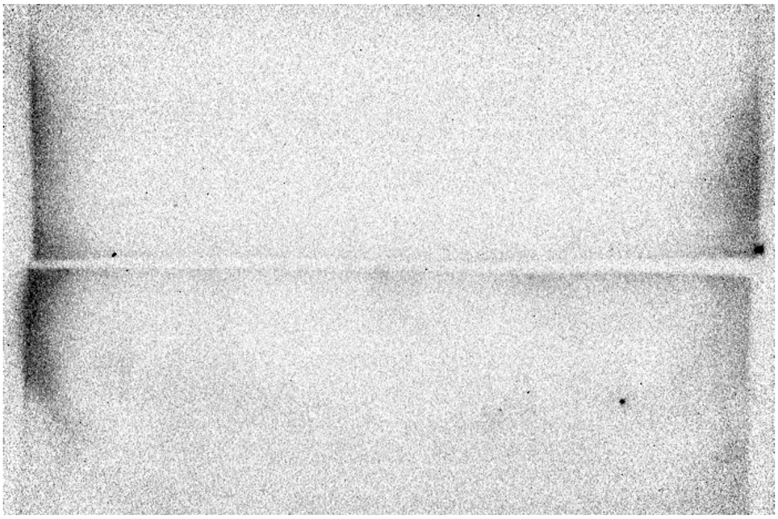

Phospho 4E-BP1 (Thr37/46)

~18 kDa

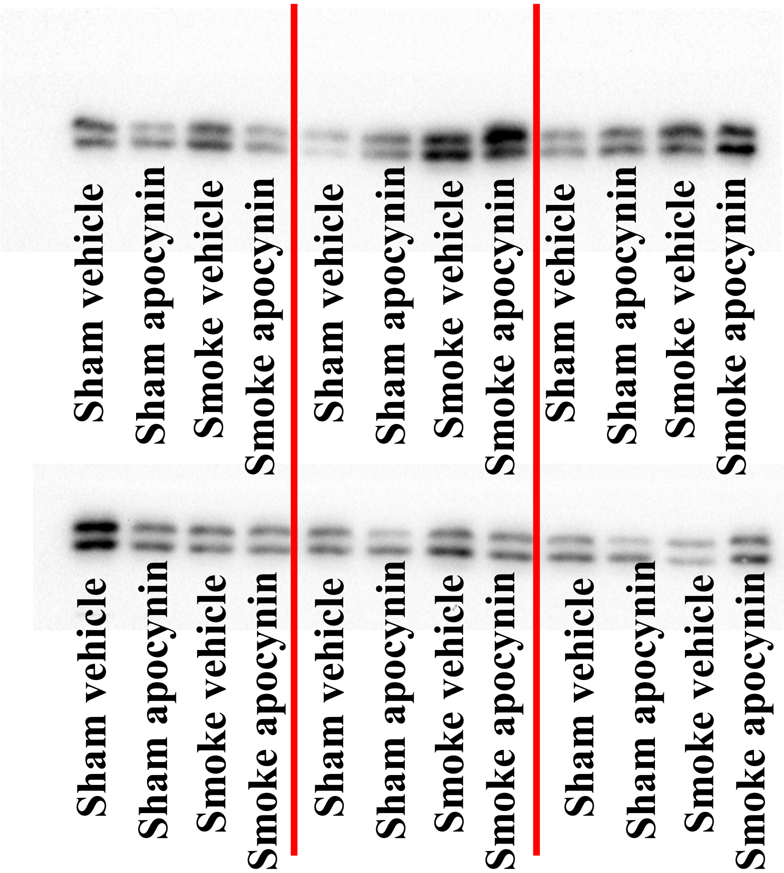

Each lane represents sample from 1 mouse

Figure S2 (continue)

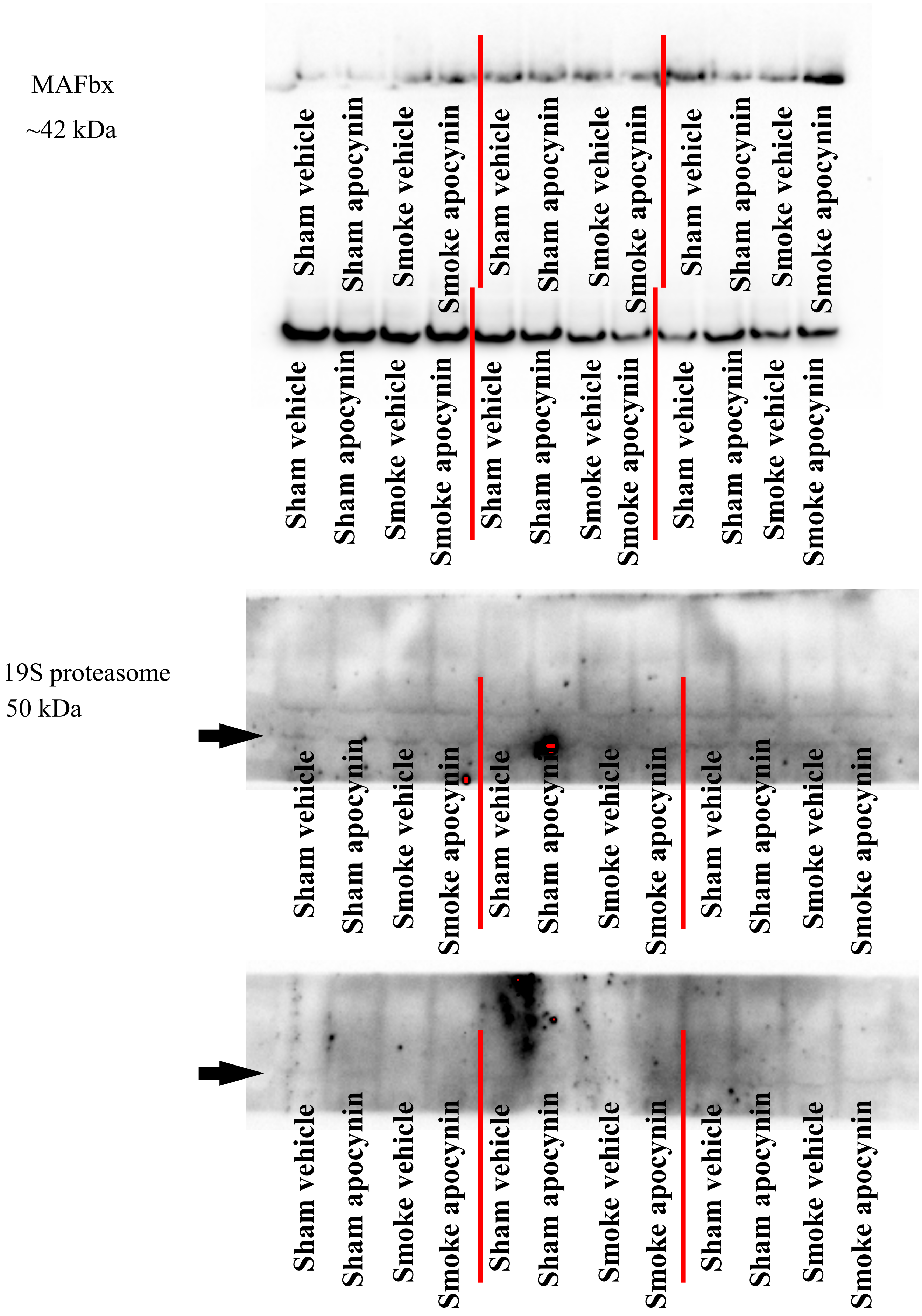

Figure S2 (continue)

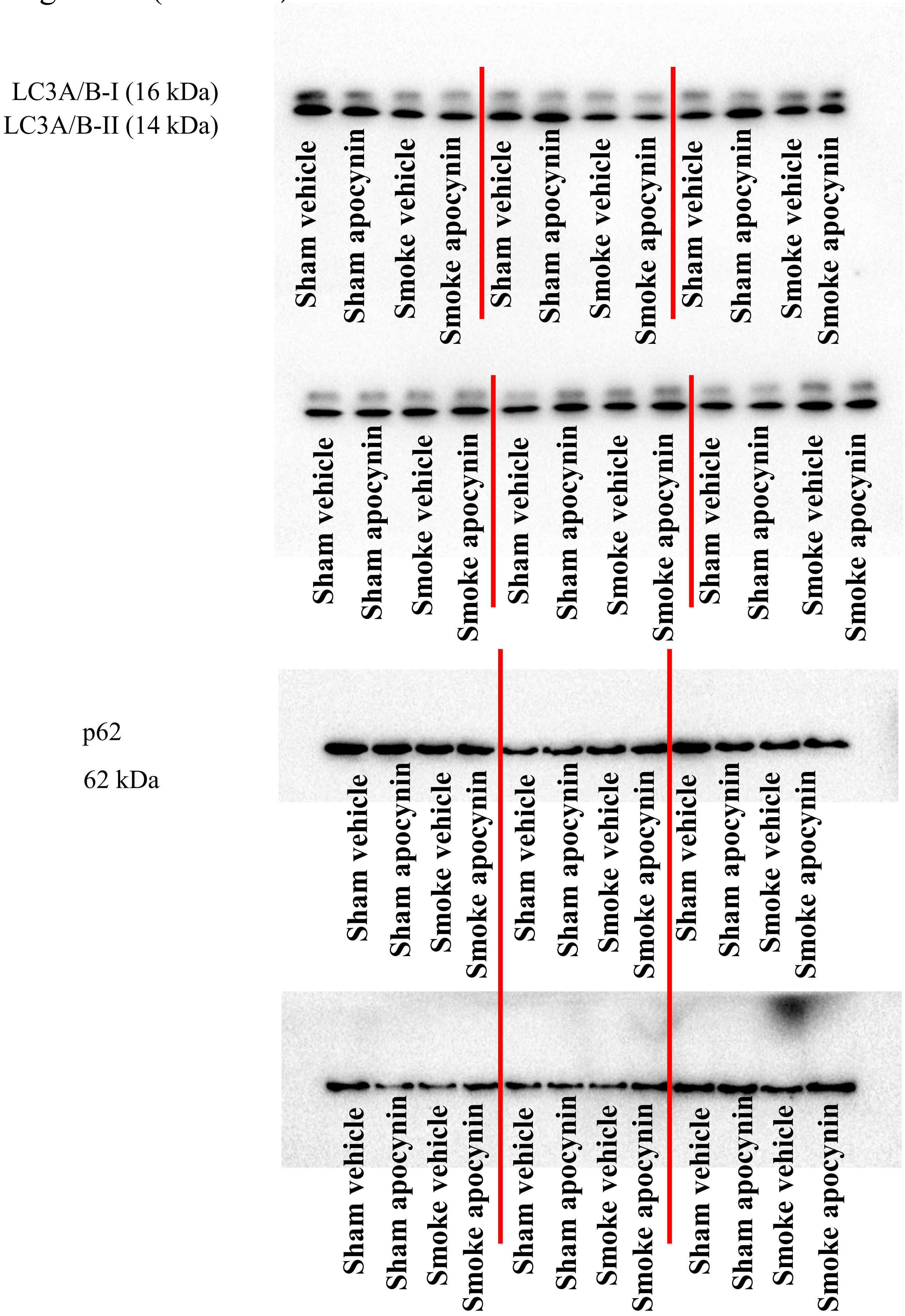

Figure S2 (continue)

GAPDH

37 kDa

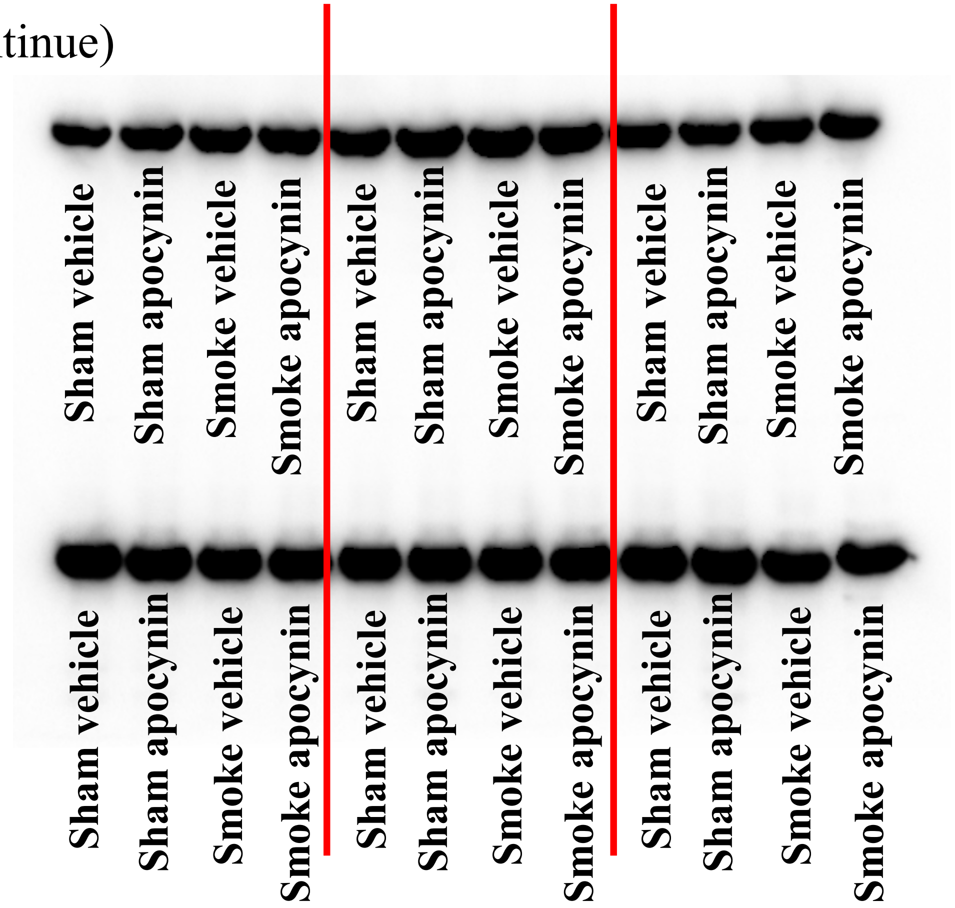

Supplement: Supplementary file 1 — Data S1. Supporting information [file BPH-178-3049-s001.pdf]
